# Challenges and best practices for recruiting families of children with intellectual disabilities for health research

**DOI:** 10.1177/17446295241255178

**Published:** 2024-05-16

**Authors:** Morgan MacNeil, Britney Benoit, Timothy Disher, Aaron J Newman, Marsha Campbell-Yeo

**Affiliations:** School of Nursing, 3688 Dalhousie University, Halifax, NS, Canada; MOM-LINC Lab, IWK Health, Halifax, NS, Canada; Rankin School of Nursing, Faculty of Science, 205191 St. Francis Xavier University, Antigonish, NS, Canada; 435677 EVERSANA, Burlington, ON, Canada; Faculty of Computer Science, 3688 Dalhousie University, Halifax, NS, Canada; Department of Pediatrics, Psychology and Neuroscience, 3688 Dalhousie University, Halifax, NS, Canada; School of Nursing, 3688 Dalhousie University, Halifax, NS, Canada; MOM-LINC Lab, IWK Health, Halifax, NS, Canada; Department of Pediatrics, Psychology and Neuroscience, 3688 Dalhousie University, Halifax, NS, Canada

**Keywords:** children, infant, intellectual disability, recruitment, research

## Abstract

Research focused on children with intellectual disabilities has been of increasing interest over the last two decades. However, a considerable lag in the amount of research that is representative and generalizable to this population in comparison to neurotypical children remains, largely attributed to issues with participant engagement and recruitment. Challenges and barriers associated with engaging and recruiting this population include lack of research to provide a sound foundation of knowledge, ethical considerations, parental attitudes, family commitments, and organizational gatekeeping. Researchers can engage children and their families using participatory research methods, honouring the child’s right to assent, and collaborating with parents. Recruitment strategies include partnering with organizations, working with parent and patient partners, and using remote methods. Employing evidence-informed engagement and recruitment strategies may provide substantial social and scientific value to the research field by ensuring that this underrepresented population benefits equitably from research findings.

## Introduction

Children with intellectual disabilities are more likely to have complex health conditions, co-morbidities, and increased needs for health services than neurotypical children (Cogswell et al., 2022; Lindly et al., 2020; Raskoff et al., 2023; Zablotsky et al., 2019). Despite this, children with intellectual disabilities are often deliberately or accidentally excluded from health research. This exclusion makes children with intellectual disabilities vulnerable to health disparities, as we lack sufficient evidence to guide their complex care (Raskoff et al., 2023). Further, the exclusion of this group can make samples unrepresentative of the pediatric population at large, limit diversity in the health experiences being studied, and diminish the relevance of research findings (Njelesani et al., 2022).

The lack of research including children with intellectual disabilities can be attributed, in part, to recruitment challenges (Cleaver et al., 2010; Lennox et al., 2005; Nicholson et al., 2013). Of the limited number of studies that are conducted focusing on children with intellectual disabilities, there has been inconsistent reporting of participation rates as well as a lack of detail on participant recruitment methods (i.e., how participants were successfully recruited) (Cleaver et al., 2010). Increasing the recruitment and enrollment of children with intellectual disabilities in health research, along with reporting how recruitment methods are undertaken in studies to assist in future recruitment efforts, could provide substantial social and scientific value by ensuring that this underrepresented population benefits equitably from research findings (Banas et al., 2019; Njelesani et al., 2022).

## Objective

This paper will discuss the various challenges involved with recruiting children with intellectual disabilities and their families for health research. Best practices surrounding engagement and recruitment of this population will also be discussed.

## Methods

A narrative review of the current literature surrounding research engagement and recruitment methods for children with intellectual disabilities and their families was conducted in January 2024 using PubMed. As the literature pertaining to this subject area is limited, no date limiters were placed on the search. See Table 1 for the search strategy that was developed in consultation with a librarian.

**Table 1.** Search strategy.

#	Query	Result
1	pediatric*[Title/Abstract] OR paediatric*[Title/Abstract] OR child*[Title/Abstract] OR infant*[Title/Abstract] OR adolescen*[Title/Abstract] OR “Young person”[Title/Abstract] OR youth*[Title/Abstract] OR teen*[Title/Abstract] OR preteen*[Title/Abstract] OR baby[Title/Abstract] OR babies[Title/Abstract] OR toddler*[Title/Abstract] OR boy*[Title/Abstract] OR girl*[Title/Abstract] OR “young people”[Title/Abstract] OR “young adult*”[Title/Abstract]	2,838,118
2	(MH “Child+”) OR (MH “Adolescence+”)	45,716
3	S1 OR S2	2,846,489
4	TI (research OR study OR recruit* OR engage* OR retain* OR stakehold* OR involve* OR community engagement OR co-design OR participatory research) AB (research OR study OR recruit* OR engage* OR retain* OR stakehold* OR involve* OR community engagement OR co-design OR participatory research)	2,236
5	TI ( (intellectual* (disab* OR disord* OR impair*) OR learning (disab* OR disord* OR impair*) OR cognit* (disab* OR disord* OR impair*) OR development* (disab* OR disord* OR impair*) OR neurologic* (disab* OR disord* OR impair*) ) OR AB ( (intellectual* (disab* OR disord* OR impair*) OR learning (disab* OR disord* OR impair*) OR cognit* (disab* OR disord* OR impair*) OR development* (disab* OR disord* OR impair*) OR neurologic* (disab* OR disord* OR impair*) )	91,413
6	TI “down syndrome” OR “prader willi syndrome” OR “cerebral palsy” OR “fragile x syndrome” OR “autism” OR AB “ down syndrome” OR “prader willi syndrome” OR “cerebral palsy” OR “fragile x syndrome” OR “autism”	125,129
7	(MH “Intellectual Disability+”)	1,600
8	S5 OR S6 OR S7	127,825
9	S3 AND S4 AND S8	70

Following a literature search of PubMed, studies were uploaded into Covidence (a web-based platform that streamlines the production of systematic and other literature reviews). Titles and abstracts, followed by full texts, were screened. The reference lists of all included articles were further screened for eligible papers.

## Recruitment challenges

### Lack of research

While research focused on children with intellectual disabilities has been of increasing interest over the last two decades, there remains a considerable lag in the amount of research involving children with intellectual disabilities as participants, and general pediatric research that is representative or generalizable to this population (Banas et al., 2019; Cleaver et al., 2010; Mash and Barkley, 2006). The extent to which children with intellectual disabilities are excluded from child developmental research across two high impact journals was examined by Feldman et al. (2013). Of the 533 articles that were included in this study, Feldman et al. (2013) found that 66.7% of articles that mentioned “disability” explicitly excluded children with varying intellectual disabilities; the rate of exclusion approached 90% when they included articles that did not mention “disability”, where the researchers assumed that no mention of the term meant they were excluded. Similarly, a scoping review by Camanni et al. (2023) explored the exclusion rate of children with disabilities in interventional clinical trials related to common disorders (e.g., diabetes, congenital defects, pneumonia) affecting pediatric populations. Of the 328 included studies, they found only one trial with “absolute” exclusion criteria, meaning the condition of the child in relation to the study purpose made it impossible for the child to participate. This evident dearth in the literature, in combination with inconsistent methods of recruitment for this population being reported, contributes to challenges with recruitment for studies focused on children with intellectual disabilities, as researchers do not have a sound foundation to guide their recruitment procedures (Cleaver et al., 2010; Ouellette-Kuntz et al., 2013).

### Ethical considerations

All children, regardless of their intellectual abilities, are considered vulnerable research subjects who require additional safeguards to protect their well-being compared to adults (Bailey et al., 2014; Damsma Bakker et al., 2021). Thus, there are ethical guidelines in place to guide their participation in clinical research (Canadian Institutes of Health Research, Natural Sciences and Engineering Research Council of Canada and Social Sciences and Humanities Research Council of Canada, 2022; European Union Agency for Fundamental Rights, 2014; U.S. Department of Health and Human Services, 2016). Children with intellectual disabilities, who are viewed as especially vulnerable, may require supplementary safeguards compared to neurotypical children (Thompson et al., 2020). Research studies such as the Willowbrook State School for Children with Mental Retardation (Krugman, 1986) and the Jewish Chronic Disease Hospital (Arras, 2008) highlight the importance of taking expanded ethical precautions when conducting research with these children to protect their vulnerability. While there are guidelines in place for conducting research with children, and separate guidelines for conducting research with persons with altered cognitive ability, there are limited guidelines available when these vulnerabilities intersect. Thus, a grey area remains for researchers and their responsibilities in conducting ethical research with children with intellectual disabilities, which may lead to deliberate or accidental exclusion to protect them from harm (Lennox et al., 2005; Thompson et al., 2020). While protection from harm is critical, there must be a harmonious balance between protection and supporting inclusivity in research to yield equitable findings (Graham et al., 2013).

Obtaining informed consent for participation in research is viewed as an ongoing process as opposed to a single moment in time (Government of Canada, 2023b). This means that parents must be aware that they have a right to withdraw at any point in time throughout the study, even if verbal or written consent has been obtained. While these points may seem widely known among parents, studies have revealed that parents were not knowledgeable of their right to withdraw from the study (Kumari et al., 2019) and could not recall the risks associated with the study (Gertsman et al., 2020) following completion of the consent process. The process of consent and assent are often insufficiently adapted to be inclusive of intellectual disabilities. Therefore, children with intellectual disabilities are frequently excluded from the opportunity to provide assent or dissent, taking away their right to express their views on matters that affect them (Thompson et al., 2020).

### Parental attitudes

Parents of children with intellectual disabilities are viewed as key components in deciding whether their child will be enrolled in a research study (Bye and Aston, 2016; Hodapp, 2007). Parental attitudes surrounding the research study are central to successful recruitment of children with intellectual disabilities (Lott et al., 2022). Often, parents of children with intellectual disabilities refuse to enroll their child in research studies before receiving all information on the purpose of the study and procedures due to fear of randomization (i.e., fearing that their child will be randomized to the control group and be put at a disadvantage), fear of finding out negative test results (i.e., biopsies, genetic testing), fear of discrimination from the healthcare team, lack of exposure to benefits of research studies involving children with intellectual disabilities, and a general mistrust of the healthcare system and research personnel (Drury et al., 2021; Idnay et al., 2023; Kaiser et al., 2017; Lebensburger et al., 2013; Lennox et al., 2005; Lott et al., 2022; Nicholson et al., 2013; White et al., 2022). Parents of children with intellectual disabilities act as their child’s primary advocate (Kruithof et al., 2020). Therefore, these parents partake in a thorough risk-benefit analysis before enrolling their child in research studies (White et al., 2022). This was demonstrated by Drury et al. (2021), where parents weighed the risks and benefits of their child having additional biopsies and follow-up bloodwork during a scheduled heart surgery. When parents were told that invasive procedures are involved in the study (e.g., bloodwork) and were provided with no additional context (e.g., the bloodwork will be done through a central line that will already be placed in the child post-heart surgery), they were likely to deny study enrollment. This is also the case when information about the study seems deceiving, there is not a clear potential benefit for the child, or a potential benefit for the intellectual disability community at large (Drury et al., 2021; Lennox et al., 2005; Nicholson et al., 2013; White et al., 2022).

Parents of children with intellectual disabilities generally have numerous stressors and demands in their lives related to their child’s diagnosis and co-morbidities, financial concerns, altered coping mechanisms, and busy schedules (Caicedo, 2014). Balancing these stressors and associated anxiety may lead to parents viewing research participation as a burden and decline to enroll their child (Hayman et al., 2001; Smyth et al., 2012; Tooher et al., 2008; Wilman et al., 2015). This concept was congruent across numerous studies included in a systematic review by Wilman et al. (2015), where parents expressed their reluctance to enroll their preterm or sick neonates in research because of the added anxiety on top of their already elevated stress levels while in the NICU. Along with the time commitment that is involved with participating in research, other factors to consider are the mental load, cost of transportation (e.g., gas or alternative fuels, public transport, parking), and feasibility of rearranging a work schedule or missing days of work (Lebensburger et al., 2013).

Parents of children with intellectual disabilities are more likely to allow their child to participate in research if there is a clear and obvious benefit for their child or the larger community of persons with intellectual disabilities (Lebensburger et al., 2013; White et al., 2022). This point was highlighted by Andrews et al. (2020), who explored the difference between parents’ views on sharing their child’s electronic health record for research purposes when their child had fragile X syndrome, autism, or was neurotypical. Parents of children with fragile X syndrome and autism were more willing to share their child’s mental health and genetic information, in contrast to parents of neurotypical children, because they felt that they could potentially help the larger fragile X syndrome and autism communities (Andrews et al., 2020). White et al. (2022) examined parental willingness to participate in research specific to Down syndrome, including their current research perceptions, willingness to participate in different types of research, and their past experiences with research participation. Parents from this study reported feeling comfortable with learning, accessing, and digesting information about research studies, and 70% reported a willingness to participate in Down syndrome focused research (White et al., 2022).

### Gatekeeping

A gatekeeper is defined as someone who is in control of who can access an institution or organization (e.g., school principal, managing director, administrator) (Ahern, 2014). Gatekeepers can also be informal (i.e., not affiliated with an organization), where their role is more related to befriending, supporting, and protecting vulnerable persons (Emmel et al., 2007). Because children with intellectual disabilities are considered a hard-to-reach population, researchers often require the cooperation and assent of gatekeepers who support or work with potential participants (Williams, 2020). This can be a significant barrier to researchers, as gatekeepers may not be enthusiastic about research studies being conducted with participants of their organizations, often related to a general mistrust of researchers (Emmel et al., 2007). Thus, gatekeepers can choose to deny researchers access to the population of interest.

When researchers partner with an organization for recruitment support, gatekeepers work above and beyond what they are expected to do as part of their regular job. This can be troublesome among gatekeepers who are overwhelmed with their regular workload, as they may feel that they do not have time to dedicate to recruiting participants (Lennox et al., 2005). When gatekeepers lack knowledge on the purpose of the study, they may feel uncomfortable approaching potential participants about recruitment. Nicholson et al. (2013), through interviewing gatekeepers about recruiting persons with intellectual disabilities, found that they did not want to partake in recruitment due to fear of introducing bias into the research and making potential participants feel pressured to participate. These fears may prevent gatekeepers from engaging in recruitment, thus leading to low enrollment. Researchers may also encounter barriers to recruitment when working through the multiple tiers of management that may be present within an organization, leading to broken communication. For example, Lennox et al. (2005) described an organization that had 17 different levels of management that needed to be reported to before engaging in recruitment strategies; as a result, no participants ended up being recruited for that study.

## Engagement and recruitment strategies

The implementation of evidence-based engagement and recruitment strategies are essential when conducting research with children with intellectual disabilities and their families. These strategies require a significant amount of both financial and administrative investment to be successful (Idnay et al., 2023).

### Parent-researcher collaboration

An effective way to engage children with intellectual disabilities is to collaborate with the child’s parents. Parents are considered experts on their child’s behaviour, needs, and interests (Bogetz et al., 2021). Therefore, parents may offer researchers helpful suggestions on how to engage their child throughout the entirety of the research process. For example, a parent may suggest that their child enjoys playing with technology, so the researcher could engage the child by allowing them to test electronic devices that will be used for an intervention in a study (Saturno et al., 2015). Parents can assist researchers in identifying behavioural patterns that reflect how the child is feeling (e.g., nervous, uncomfortable, disengaged, etc.), which are important cues to be aware of for successful engagement (Carroll and Sixsmith, 2016).

Participatory research is a person-centered engagement method that is employed by researchers to prioritize participants as equal partners, experts, and as researchers themselves (Wintels et al., 2018). Participatory research is an iterative process of producing knowledge and co-learning, where the person with lived experience is placed at the center of the process (Kesby et al., 2007). Using participatory research is a valuable engagement strategy among children with intellectual disabilities, as they have historically not been viewed as equals with neurotypical society, nor had the opportunity to collaborate and share their opinions (Heller et al., 2005). Examples of participatory research engagement strategies to use with children with intellectual disabilities include using a variety of data collection methods (e.g., pictures, observations), conducting interviews with the child, and carefully selecting data collection methods based upon the child’s level of development and preference (Feldner et al., 2019; Wintels et al., 2018). Danker et al. (2017) found photovoice (i.e., the use of photographs as prompts to facilitate discussions during interviews) to be a successful engagement strategy among children with autism. Because photovoice requires less eye contact between the interviewer and participant, which is a social skill people with autism may struggle with, photovoice was deemed an important engagement strategy to use with this population. Further, in this study researchers empowered the children by allowing them to lead the interviews and discussions (e.g., allowed them to handle the tablet, select what photographs they wanted to discuss in what order), which led to additional positive engagement.

Participatory research can also be used as an engagement strategy with parents of children with intellectual disabilities. This can be done by including parents as members of the research team to inform all aspects of the study and collaborating with parents to co-design study materials and protocols, collect and analyze data, and inform policy changes (Wikman et al., 2018). Nguyen et al. (2024) created a Patient and Family Advisory Council (PFAC) to engage adolescents, young adults, and parents/caregivers as research partners to develop an App to support transitioning from pediatric to adult care. Feedback received from the PFAC shaped the researchers approach to establishing engaging partnerships over the course of the App design. Their engagement priorities included: 1. Offering clarity and flexibility around participation, 2. Valuing and acknowledging partners and their contributions, and 3. Providing choice and leveraging individual interests and strengths.

Positive, trusting, collaborative relationships between researchers, clinicians, and participants are associated with more favourable perceptions of research and acceptability (Drury et al., 2021). Building trust can be accomplished by fostering an environment that prioritizes partnership with the parents and participants as opposed to a hierarchical dynamic, having the primary investigator of the project or the research coordinator invite parents to be part of the research team, meeting with the family face-to-face to build rapport and answer questions, and providing the parents with clear information about the study (Banas et al., 2019; Barnes, 2003; Drury et al., 2021; Kavanaugh et al., 2006; Knox and Burkhart, 2007). Nicholson et al. (2013) suggested that having the researcher familiarize themselves with potential participants through informal meetings at support groups, medical centres, and organizations can assist with reducing anxiety and building trust, leading to higher study enrollment. This can also be accomplished through more remote interventions such as creating videos about the study and sharing them with parents and their children, so that they can hear the information repeatedly and familiarize themselves with the researcher in the video and study environment (Nicholson et al., 2013).

Using multiple recruitment modalities (e.g., face-to-face meetings, written material, videos) increases inclusiveness and accessibility within this population, as individuals who have hearing or visual impairment can still participate in learning about the study. Further, this supports the competing demands that families of children with intellectual disabilities have, as it demonstrates a flexible recruitment approach. Additional measures that can be taken to accommodate parents with competing demands include visiting the family’s home to discuss the study and working before or after hours to complete data collection or recruitment in a way that suits the family’s schedule (Lennox et al., 2005). Offering stipends, for participation on research teams, and for transportation and food costs may also help in recruiting and retaining parents with tight schedules and limited finances (Novak-Pavlic et al., 2023).

### Clear communication

When communicating with parents, researchers must take the time to explain the risks and benefits of study participation clearly. This can include how the child may directly benefit from study participation, or how the intellectual disability community at large may benefit, both of which are important pieces of information for parents (Andrews et al., 2020; White et al., 2022).

When information about the study is being provided to parents, it is important to limit the use of jargon and complex terms to ensure that parents have a sound understanding of what is involved in the research study (Kaiser et al., 2017). Lebensburger et al. (2013) found that African parents of children with sickle cell anemia did not want their child to participate when they heard the term “research study”, as they were fearful that their child would be treated like a lab guinea pig. Along with this, researchers should ensure they are using inclusive, sensitive, and active language, and limit the use of passive language (Government of Canada, 2023a; Kavanaugh et al., 2006). For example, saying “your child” as opposed to “the child”, and “you will be asked several questions” as opposed to “the researcher will ask questions” (Kaiser et al., 2017; Kavanaugh et al., 2006).

Thoughtful use of language is also important to include in any written materials that are provided to the parents or child, as these materials are often their first introduction to the research study. Therefore, researchers must be cognizant of the words, graphics, and designs that are used in written materials. This was eloquently demonstrated by Kavanaugh et al. (2006), who thoughtfully designed an abstract photo (i.e., the infant was not clearly identifiable as an extremely preterm infant) of a mother and preterm infant to use in their recruitment materials, as opposed to a generic photo of a mother and infant, to convey hope and realistic optimism among mothers who birthed or were expected to birth preterm infants. These careful considerations in language execution and design, while seemingly subtle, can have a substantial impact on parental decision making in relation to study enrolment (Government of Canada, 2023a). These language considerations are also important aspects of establishing and maintaining trust with parents and children with intellectual disabilities throughout the recruitment and study process (McDonald et al., 2022). When communicating with parents, researchers must not be presumptuous in thinking that parents of children with intellectual disabilities are stressed, depressed, or disappointed with their child’s diagnosis. If researchers inaccurately make these assumptions and allude to these feelings through their recruitment approach, they may inadvertently put these negative connotations into the parents’ heads (Kavanaugh et al., 2006). Thus, part of clear communication is approaching each potential participant in an individualized manner and meeting them where they are at in their lives. Seeking input from parents involved as members of research teams, or from partnering parent organization can be an important strategy in ensuring appropriate language is used.

### Obtaining consent and assent

While it is the parent’s responsibility to consent for their child to be enrolled in a research study, the process of obtaining assent from the child with an intellectual disability should not be disregarded. Allowing the child the opportunity to assent or dissent places them in an autonomous and powerful position. Providing the child with an opportunity to ask questions and share their thoughts supports their ability to make decisions that affect them, respects their role in the research process, and allows them to be taken seriously; all of which help engage the child in the research (Garth et al., 2009; Gonzalez et al., 2021). In a study examining parents of children with fragile X syndrome and their ratings of their child’s ability to participate in the assent process (not capable at all, capable with a lot of support, capable with minimal support, fully capable), 32% of males and 74% of females had no items rated as “not capable at all”, suggesting that there is a wide range of ability and potential for these children to provide assent, according to their parents (Bailey et al., 2014). Therefore, instead of researchers assuming the child cannot meaningfully contribute to conversations about consent and assent because they have an intellectual disability, researchers should first discuss the child’s ability to partake in the conversation with the parents. When children with attention-deficit/hyperactivity disorder were not included in the consent/assent process, they were more likely to refuse to participate and drop out of the study later (Gray, 2017). Thus, attempting to include the child with an intellectual disability in the consent and assent process with their parents is important for making the child feel like an equal member of the research team, and helps with sustained enrollment.

### Organizational partners

Locating potential participants for a research study can be a challenge. Working through organizations and intermediaries (i.e., family members, healthcare personnel, teachers, etc.) can provide an accessible route to establish contact with hard-to-reach populations (Lennox et al., 2005; Nicholson et al., 2013). Forming early connections with organizations and intermediaries can facilitate successful recruitment for research studies (Lennox et al., 2005). Having trusted members of organizations and intermediaries reach out to potential participants has proven to be a more successful recruitment method than traditional recruitment methods, such as telephoning potential participants (Lennox et al., 2005). These organizations can recruit potential participants through handing out recruitment forms, sending mass emails, telephoning, or sending paper mail (Ouellette-Kuntz et al., 2013). Partnering with support groups that parents of children with intellectual disabilities access has been coined as “parent-centered recruitment” and has proven to be a successful recruitment strategy for parents of children with autism (Zamora et al., 2016). Partnering with various community groups and organizations can be of particular benefit when attempting to recruit a culturally diverse sample, as many ethnic minority individuals access support services through these groups (Gonzalez et al., 2021; Zamora et al., 2016).

As previously discussed, organizational members may gatekeep potential participants and information from researchers. To mitigate this, researchers must focus on building trusting relationships with intermediaries to support recruitment efforts for hard-to-reach populations. This can be accomplished through establishing a personal relationship with intermediaries and answering any questions they may have about the study to reduce confusion and anxiety (Lennox et al., 2005). Because recruitment techniques are not required skills that intermediaries must have, they may benefit from specific training and resources provided by researchers to facilitate recruitment efforts (Lennox et al., 2005). This was alluded to in a study by Nicholson et al. (2013), when an intermediary stated that if she had been given specific advice and strategies on how to actively recruit potential participants, as opposed to just handing out flyers about the study, the recruitment process would have been more successful. To encourage intermediaries to engage in recruitment efforts, researchers must express the importance of the bigger “why?” for the research, as well as educating the intermediary on their influential position in securing enrollment into the study, and the importance of studies on persons with intellectual disabilities (Lennox et al., 2005; Nicholson et al., 2013).

Partnering with clinical research registries is another way that researchers can recruit persons with intellectual disabilities. Clinical research registries are organizations that provide persons with intellectual disabilities and their families the opportunity to be contacted by researchers for participation in studies that they meet eligibility criteria for (Kalb et al., 2019). These organizations are often of interest to researchers due to their low cost and proactive approach to study recruitment (Kalb et al., 2019). Because it is the responsibility of the participant to sign up for a registry, researchers can play a pivotal role in providing registry information and encouraging families to sign up.

### Parent and patient partners

Parent partners have been playing an increasingly large role as collaborators and advisors on the research team to help with approaching families, designing study materials, and communicating with families throughout the project (Curran et al., 2018; Domecq et al., 2014; Flynn et al., 2019; Hartling et al., 2021; Kim et al., 2023; Vanstone et al., 2023). Working with parent partners to help with both the design and conduct of research studies can be helpful for overcoming barriers to recruitment that exist among hard-to-reach populations (Janvier et al., 2019; Kaiser et al., 2017). Kavanaugh et al. (2006) demonstrated the value of working with a parent partner in their study when they had a mother of a child born prematurely review all study materials before they were disseminated among mothers who were expected to birth a premature infant. In addition to assisting with the design of study materials, parent partners are also important for setting priorities for clinical and empirical research (Sacristan et al., 2016).

Having parent partners assist with recruiting participants is of particular importance among families of children with intellectual disabilities. Parent partners can assist with recruitment by sharing recruitment materials through their personal networks (Flynn et al., 2019). When approaching potential participants, parent partners can empathize with fellow parents of children with intellectual disabilities on the stress, uncertainties, and complexities that accompany their role as a parent, and thoughtfully relay this empathy through recruitment materials (Sartore et al., 2021). Parent partners are valuable resources in assisting with the sensitive approach that must be used when recruiting potential vulnerable participants, such as high-risk neonates (Janvier et al., 2019). Parent partners express being motivated to partner with researchers to assist in the design, conduct, and recruitment for studies due to personal connections that they feel with the study topic (Kim et al., 2023).

Researchers can also partner with persons with intellectual disabilities (i.e., patient partners) to facilitate study design and recruitment methods. Historically, the involvement of persons with intellectual disabilities on the research team itself has not been considered best practice (Barnes, 2003). However, this method is increasingly becoming of interest as researchers incorporate the principles of equity, diversity, and inclusion into their methodologies, along with a compliance to the central ethos of self-advocacy – “nothing about us without us” (Frawley and Bigby, 2014; Government of Canada, 2023a; St. John et al., 2018). Partnering with persons with intellectual disabilities, which is commonly termed “inclusive research”, to assist in recruiting families of children with intellectual disabilities can be advantageous as their life experience as an individual with an intellectual disability can help shape the purpose and specific benefits of the research (Bigby et al., 2014; Leech, 2004). Gary et al. (2012) emphasized that it is important to have persons with intellectual disabilities involved in the research design and recruitment process from the beginning because it shows that the study matters to them and may impact their lives in a way that is different from neurotypical research team members. The shared experience of intellectual disability between patient partners and potential research participants can contribute a newfound depth and richness to findings yielded from studies (St. John et al., 2018).

### Remote recruitment

Social media platforms have proven to be productive recruitment strategies over the last few decades due to their cost-effectiveness, promptness, and wide-reaching ability (Darko et al., 2022). Social media recruitment strategies can include posting advertisements for the study on Instagram, Facebook, X, Reddit, and videos on YouTube. Using social media to recruit hard-to-reach families can be of particular benefit, as these families are often busy with their child’s complex care needs, or other family matters (Johnson et al., 2019). A literature review by Darko et al. (2022) looking at social media use for research recruitment found that 38% of the studies included for the review involved using social media as a recruitment strategy specifically for hard-to-reach populations. Tan et al. (2021) found social media to be an accessible recruitment strategy for parents and caregivers of children with cancer and complex needs. Having an advertisement on social media may be an easy way to recruit these families as opposed to relying on them attending a support group or organization (Darko et al., 2022; Johnson et al., 2019).

## Conclusion

Conducting research focused on children with intellectual disabilities is increasingly being recognized as an important topic. Because this population has a history of being excluded from research, population-specific engagement and recruitment strategies must be employed, including consulting with parents, participatory research, partnering with organizations and intermediaries, using remote methods, and partnering with parent and patient partners. While these strategies may seem transferrable to neurotypical pediatric populations, this work adds specific information for children with intellectual disabilities, who are the most underrepresented in research. Future research should be dedicated to prioritizing methods of engagement and recruitment, investigating the feasibility of these methods in different research contexts, and developing assent guidelines specific to conducting research with children with varying intellectual disabilities. When time and finances are dedicated to engagement and recruitment strategies for this vulnerable population, and research findings at large become more representative of this population, substantial social and scientific value is added to the research field by ensuring that there is equitable benefit from research findings.
